# Structure-Specific DNA Endonuclease Mus81/Eme1 Generates DNA Damage Caused by Chk1 Inactivation

**DOI:** 10.1371/journal.pone.0023517

**Published:** 2011-08-17

**Authors:** Josep V. Forment, Melanie Blasius, Ilaria Guerini, Stephen P. Jackson

**Affiliations:** The Gurdon Institute and the Department of Biochemistry, University of Cambridge, Cambridge, United Kingdom; Universita' di Milano, Italy

## Abstract

The DNA-damage checkpoint kinase Chk1 is essential in higher eukaryotes due to its role in maintaining genome stability in proliferating cells. *CHK1* gene deletion is embryonically lethal, and Chk1 inhibition in replicating cells causes cell-cycle defects that eventually lead to perturbed replication and replication-fork collapse, thus generating endogenous DNA damage. What is the cause of replication-fork collapse when Chk1 is inactivated, however, remains poorly understood. Here, we show that generation of DNA double-strand breaks at replication forks when Chk1 activity is compromised relies on the DNA endonuclease complex Mus81/Eme1. Importantly, we show that Mus81/Eme1-dependent DNA damage—rather than a global increase in replication-fork stalling—is the cause of incomplete replication in Chk1-deficient cells. Consequently, Mus81/Eme1 depletion alleviates the S-phase progression defects associated with Chk1 deficiency, thereby increasing cell survival. Chk1-mediated protection of replication forks from Mus81/Eme1 even under otherwise unchallenged conditions is therefore vital to prevent uncontrolled fork collapse and ensure proper S-phase progression in human cells.

## Introduction

Mouse embryos devoid of the DNA-damage checkpoint kinase Chk1 show pre-implantation lethality due to a severe proliferation defect [1], [2]. In addition, *CHK1* gene deletion in adult proliferating tissues [3] or Chk1 inhibition in human tissue culture cells [4] causes cell-cycle defects associated with DNA-damage accumulation in S phase that eventually lead to cell death. It has been shown that cell-cycle deregulation in Chk1-deficient cells occurs at least in part via unscheduled increases in cyclin-dependent kinase (CDK) activity due to stabilization of Cdc25A, a phosphatase that activates CDKs [5], [6]. This increased CDK activity in checkpoint-deficient cells causes activation (“firing”) of replication origins that are not normally used [4], [7], and also results in premature chromatin condensation and unscheduled entry to mitosis [3]. In turn, increased origin firing markedly perturbs replication dynamics, the most obvious effect being a dramatic reduction in replication-fork progression that eventually leads to replication-fork collapse [6], [8], [9]. Fork collapse has been proposed to be the main source of the S-phase specific DNA damage that occurs upon Chk1 inhibition, a notion that is supported by the fact that this damage is replication dependent and CDK dependent [4], [10]. Whether it is cell-cycle deregulation or the appearance of DNA damage that is the prime cause of the lethality observed in Chk1-deficient cells, however, remains uncertain.

Chk1 and its activating kinase ATR protect replication forks from collapsing even under conditions where replication is not challenged by genotoxic drugs [11], [12]. However, the actual cause of replication-fork collapse when the ATR/Chk1 pathway is compromised in vertebrate cells is currently unknown. Mus81 and its binding partner Eme1 form a structure-specific 3′-flap DNA endonuclease that can process substrates resembling replication forks [13], [14], and work in fission yeast has implicated this nuclease in cleaving replication forks in the absence of an S-phase checkpoint [15], [16]. By contrast, replication-fork processing in checkpoint-deficient budding yeast requires Exo1 [17], an exonuclease also involved in DNA-end resection [18]. Here, we show that depleting Mus81 or Eme1 in human cells allows S-phase progression when Chk1 activity is compromised. Moreover, Mus81/Eme1 depletion, but not Exo1 absence, prevents DNA double-strand break (DSB) accumulation and ensuing cell death caused by Chk1 depletion or inhibition. These findings thus highlight a role for the DNA-damage checkpoint pathway in controlling nucleases to promote replication-fork stability and completion of S phase during normal cell-cycle progression.

## Results

### Chk1 inhibition by AZD7762 causes DNA damage in a Mus81-dependent manner

**Figure 1:**
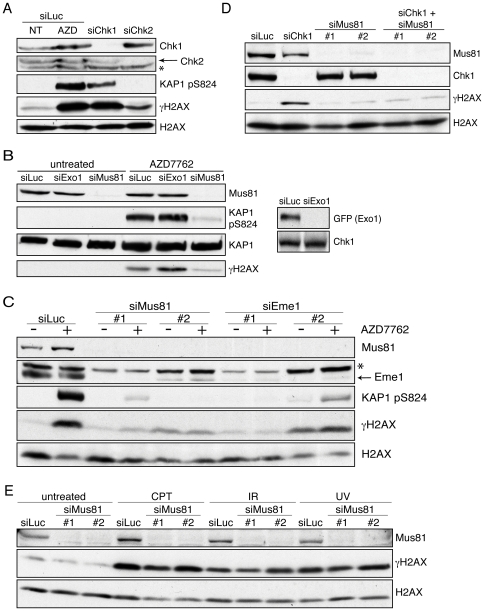
Mus81/Eme1 depletion reduces DNA-damage after Chk1 inhibition. *A.* Chk1 inhibition causes DNA damage. Cells were transfected with control siRNA (siLuc) or siRNAs targeting Chk1 or Chk2. siLuc cells were untreated (NT) or treated (AZD) for 5 h with 200 nM AZD7762. * Cross-reacting band. *B.* Mus81 but not Exo1 is involved in the generation of DNA-damage signals after Chk1 inhibition. Cells were transfected with siLuc, siExo1, or siMus81#1, and treated as in A. Due to an inability to detect endogenous Exo1, the right panel shows depletion of recombinant GFP-Exo1 using the same siRNA. *C.* Mus81 or Eme1 depletion reduces DNA-damage signals caused by AZD7762 treatment. Cells were transfected with siLuc or with siRNAs targeting Mus81 or Eme1, and treated as in A. * Cross-reacting band. *D.* Cells were initially transfected with siLuc or siMus81#1 or #2, then 24 h afterwards they were transfected with siChk1 or siLuc. Cells were collected 48 h after the second transfection. *E.* Mus81 depletion does not reduce γH2AX production after treatment with various DNA-damaging agents. Cells were transfected with siLuc or siMus81 then treated with 1 µM camptothecin (CPT), ionizing radiation (IR, 10 Gy) or ultraviolet light (UV, 10 J/m^2^), and collected 1 h afterwards.

*CHK1* gene deletion or Chk1 inhibition trigger DNA damage in replicating cells [3], [4]. To explore the mechanism for this, we used AZD7762, an ATP-competitive inhibitor of the checkpoint kinases Chk1 and Chk2 [19]. As shown in Figure 1A, treating human U2OS cells with AZD7762 caused accumulation of DNA damage, as indicated by phosphorylation of KAP1 Ser-824 and histone variant H2AX Ser-139 (γH2AX), protein targets whose phosphorylation is mediated by the DNA-damage sensor kinase ATM and by ATM plus the related kinases ATR and DNA-PK, respectively [20]. Confirming the role of Chk1 inhibition in the generation of the DNA damage, such phosphorylations were also markedly induced by short-interfering RNA (siRNA)-mediated depletion of Chk1 but not Chk2 (Fig. 1A).

Given that Chk1 inhibition dramatically perturbs normal replication-fork progression [8], [9], and since Mus81 and Exo1 are involved in processing replication forks in checkpoint-deficient yeast cells [15], [17], we tested whether human Mus81 or Exo1 might generate DNA damage following AZD7762 treatment. Strikingly, depletion of Mus81 but not Exo1 markedly reduced γH2AX and phospho-KAP1 Ser-824 signals after AZD7762 treatment (Fig. 1B). Furthermore, treating cells with several different siRNAs targeting Mus81 or Eme1 reduced γH2AX and phospho-KAP1 Ser-824 generation by AZD7762 (Fig. 1C; note that the stabilities of Mus81 and Eme1 are interdependent). Such effects were not cell-type specific, as similar findings were obtained with human HeLa and SV40-transformed MRC-5 cells (data not shown). In addition, Mus81 depletion reduced γH2AX signals when Chk1 was depleted by siRNA treatment (Fig. 1D). By contrast, Mus81 depletion did not reduce γH2AX production when cells were treated with the DNA topoisomerase I inhibitor camptothecin (CPT), ionizing radiation (IR), or ultraviolet light (UV; Fig. 1E), arguing that Mus81 depletion does not seem to have a general effect on the DNA-damage response. Taken together, these results showed a specific involvement of the Mus81/Eme1 DNA endonuclease in the generation of DNA damage caused by Chk1 inactivation.

### Mus81 depletion increases replication-fork stability and allows S-phase progression in checkpoint-deficient cells

**Figure 2:**
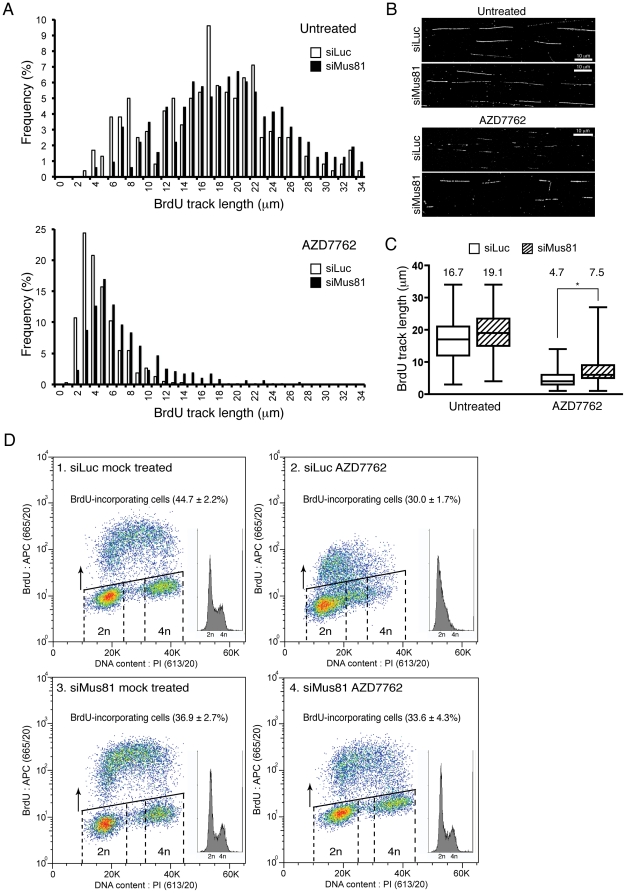
MUS81 depletion allows S-phase progression when Chk1 is inactive. *A.* Distribution of BrdU-labelled track lengths in non-treated (top panel) or AZD7762-treated cells (bottom panel). Track lengths were calculated with ImageJ software (NIH). *B.* Representative images of BrdU-stained replication tracks of non-treated (top) or AZD7762-treated cells (bottom). DNA fibres were visualized with YOYO-1 stain (Invitrogen) to discard broken fibres (not shown). *C.* Boxplot representation of the distributions of BrdU-labelled track lengths. Average track length values are denoted on top of each boxplot (untreated: siLuc *n* = 347, siMus81 *n* = 313; AZD7762-treated: siLuc *n* = 381, siMus81 *n* = 414). * Denotes statistically significant difference (*p* value<0.0001) as calculated using the non-parametric Mann-Whitney test. *D.* Flow cytometry of replicating cells as measured by BrdU incorporation. The x-axis shows DNA content by propidium iodide (PI) staining; the y-axis measures BrdU incorporation by fluorescence produced with anti-BrdU antibodies. Graphics show representative images for each experiment. Insets show histograms obtained from the same samples. Percentages were calculated from three independent experiments (± SEM). Plots and quantifications were with FlowJo 9.0.2 software (Tree Star). Cells were transfected with siLuc or siMus81#2 and treated as in Fig. 1A.

To test whether the Mus81-dependent DNA-damage formation observed upon Chk1 inactivation had any impact on replication dynamics in Chk1-inhibited cells, DNA-fibre spread experiments were performed. As the stabilities of Mus81 and Eme1 were interdependent (Fig. 1C), for this and subsequent experiments we used siRNAs directed against Mus81 and henceforth refer to the Mus81/Eme1 complex as MUS81. We depleted or mock-depleted cells of MUS81, pulsed them with the nucleotide analogue BrdU, and then assessed fork progression by measuring the lengths of BrdU-labelled tracks on DNA fibres detected by immunofluorescence. Notably, labelled track length distribution (and hence average track length) in non-AZD7762 treated cells was not significantly affected by MUS81 status, revealing that MUS81 depletion alone does not impair replication-fork progression (Fig. 2A–B top panels, and Fig. 2C). In agreement with previous reports [8], [9], we observed that inhibiting Chk1 in control cells drastically reduced the distribution of track lengths and caused the accumulation of very short BrdU tracks, indicative of impaired replication-fork processivity (compare siLuc control cells in Figs. 2A–B; track length ranges under control and AZD7762 treatment conditions are 3–35 µm and 1–13 µm, respectively). Strikingly, MUS81 depletion partially alleviated the AZD7762-induced replication defects, as observed by the fact that these cells displayed an average track length that was 60% higher than that of AZD7762-treated control cells (Fig. 2C). These results thus indicated that MUS81 is detrimental for replication-fork progression when Chk1 is inhibited.

Having found impaired replication-fork processivity in Chk1-deficient cells, we anticipated that this would have a significant impact on cell proliferation. To explore whether MUS81 depletion might affect cell-cycle progression of Chk1-inhibited cells, we used flow cytometry to analyze BrdU incorporation into cells by DNA replication. As shown in Figure 2D (compare panels 1 and 2), AZD7762 treatment of control cells caused the accumulation of cells with DNA contents between 2n and 4n, indicating an increased S-phase population (note the reduction of 4n cells). Furthermore, AZD7762 treatment also reduced the proportion of BrdU-incorporating cells, indicating decreased replication. In agreement with results obtained with DNA-fibre spreads (Fig. 2A–C), treating mock-depleted cells with AZD7762 also reduced the intensity of BrdU incorporation, reflecting reduced rates of replication-fork progression (Fig. 2D, compare panels 1 and 2). By contrast, treating MUS81-depleted cells with AZD7762 only slightly reduced the proportion of BrdU-incorporating cells and did not appreciably change the intensity or distribution of BrdU incorporation (Fig. 2D; compare panels 3 and 4). Similar results were obtained when MUS81 depletion was performed in cells treated with an siRNA against Chk1 or when Chk1 was inactivated by CEP-3891, a Chk1 inhibitor that is reported not to target Chk2 [21] (Fig. S1). Collectively, these results established that MUS81 is needed for Chk1 inhibition to trigger impaired S-phase progression. Furthermore, because AZD7762 inhibits both Chk1 and Chk2, these data indicated that the inability of Chk1-deficient cells to progress through S phase does not reflect the induction of a classical checkpoint response, in agreement with earlier observations in ATR- and ATM-deficient mouse cells [11]. Instead, our results suggested that, in the absence of a functional checkpoint, MUS81-dependent DNA damage physically blocks S-phase progression.

### MUS81 depletion reduces DSB formation and increases cell survival after Chk1 inhibition

**Figure 3:**
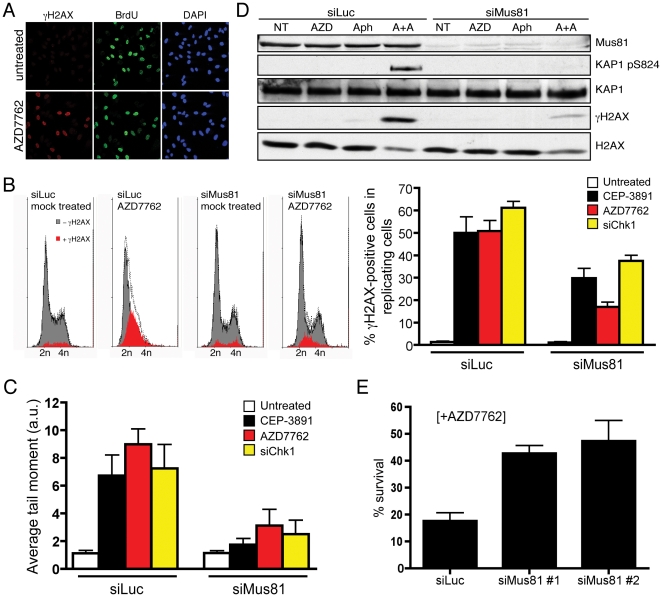
MUS81 causes DNA double-strand breaks upon Chk1 inhibition that result in cell death. *A.* AZD7762 treatment produces γH2AX in replicating cells. Cells were treated as in Fig. 1A and pulsed with 10 µM BrdU 10 min before fixation. *B.* MUS81 depletion results in reduced DNA-damage accumulation in S phase in Chk1-deficient cells. Samples were the same as in Fig. 2D and Fig. S1. γH2AX-positive cells are plotted in red and in grey are the remaining cells after the γH2AX-positive cells were subtracted (dotted line) from the total cell-cycle profile (*left panel*). Quantification of γH2AX-positive cells was performed on BrdU-positive cells to minimize effects of MUS81 depletion on BrdU incorporation (*right panel*). Plots and quantifications were with FlowJo 9.0.2 software (Tree Star). Quantifications represent means of three independent experiments (± SEM). *C.* MUS81 depletion decreases DNA double-strand break formation in Chk1-deficient cells. Neutral comet assays were performed on cells transfected with siLuc or siMus81 #2 and then treated with 200 nM AZD7762 or 500 nM CEP-3891 for 5 h, or transfected with siChk1 as in Fig. 1D. Comet analyses were performed with the CometScore software (TriTek) and average tail moments were calculated by counting approximately 100 cells for each sample. Each bar represents the average of three independent experiments (± SEM). *D.* Replication forks are likely substrates for MUS81 when Chk1 is inhibited. Cells were transfected as in Fig. 2, and left untreated (NT) or treated with 50 nM AZD7762 (AZD), 2.5 µM aphidicolin (Aph), or a combination of both drugs (A+A) for 12 h. *E.* MUS81 depletion increases cell survival after AZD7762 treatment. Cells were transfected as in Fig. 1E and treated with 200 nM AZD7762 for 24 h. Cells were next washed and incubated in normal medium, and 12 days afterwards colonies were stained with crystal violet and counted. Each bar represents the mean of three independent experiments (± SEM).

Through assessing γH2AX generation in relation to cellular BrdU incorporation by microscopy (Fig. 3A) and flow cytometry (Fig. 3B), we found that DNA damage generated by Chk1 inactivation occurred specifically in S-phase cells and was mainly MUS81 dependent. As Chk1 inhibition resulted in phosphorylation of KAP1 Ser-824 (Fig. 1A), a hallmark of DNA DSBs triggering ATM activation, this suggested that Chk1 inhibition lead to MUS81-dependent DSB formation. In line with this idea, neutral comet assays and pulse-field gel electrophoresis revealed that, while Chk1 inactivation produced marked chromosomal fragmentation in mock-depleted cells, this was significantly reduced in MUS81-depleted cells (Fig. 3C and Fig. S2). Collectively, these results indicated that DNA-damage signalling upon Chk1 inhibition mainly arises through MUS81-dependent generation of DSBs during DNA replication.

MUS81 has been implicated in the generation of DSBs at replication forks when mouse cells are treated chronically with the DNA-polymerase inhibitor aphidicolin, the ribonucleotide reductase inhibitor hydroxyurea (HU) or the DNA cross-linking agent mitomycin C (MMC). Under such conditions, MUS81-dependent DSB generation only takes place after prolonged drug treatments, and it has been shown to be important for break-induced replication fork re-start. Consequently, Mus81-deficient cells are hypersensitive to chronic treatment with these chemicals [22], [23]. On the other hand, ATR has been shown to play an important role in protecting replication forks from collapsing when cells are subjected to acute aphidicolin treatment, a function that has been suggested to be exerted through Chk1 [11]. To address whether MUS81-dependent DSBs in Chk1-deficient cells arise as a consequence of lack of replication-fork protection, we used low doses of aphidicolin to induce mild replication stress. Notably, while treating control cells with low doses of AZD7762 or aphidicolin did not induce detectable DNA-damage signals, such signals became evident when the drugs were combined, indicating that replication forks stalled by aphidicolin collapsed in the absence of active Chk1. These DNA-damage signals were, however, substantially reduced upon MUS81 depletion (Fig. 3D). Collectively, these results indicated that replication forks become substrates of MUS81 when Chk1 activity is compromised, a fact that could help explain the detrimental effect that MUS81 has on cell-cycle progression upon Chk1 inhibition. Consistent with this idea, we found that MUS81 depletion reduced cell killing by AZD7762 treatment, as measured by clonogenic survival assays (Fig. 3E).

## Discussion

We have shown that depleting the structure-specific DNA endonuclease MUS81 substantially suppresses the replication-associated effects of Chk1 inhibition on human cells. Specifically, we have established that MUS81 depletion largely prevents the generation of DNA damage caused by Chk1 depletion or Chk1 inhibition, reduces the effects of Chk1 inactivation on DNA replication and cell-cycle progression, and also prevents DSB generation when Chk1 activity is compromised. These data and the fact that MUS81 depletion partially protects cells from AZD7762-induced cell killing also imply that MUS81-dependent DSB generation is the main cause of replication failure in Chk1-deficient cells.

One of the main phenotypes observed in cells depleted of Chk1 is a marked reduction in replication-fork processivity. This is primarily due to increased CDK activity caused by stabilisation of the CDK-activating phosphatase Cdc25A upon Chk1 inhibition, which results in increased origin firing. Accordingly, inactivation of Cdc25A or inhibition of CDK activity in Chk1-deficient cells restores normal replication-fork progression and reduces the S-phase dependent DNA damage [6], [10], [24]. Although we observed a marked reduction of the S-phase dependent DNA damage in Chk1-deficient cells upon MUS81 depletion, absence of MUS81 did not fully restore replication-fork processivity (compare distributions of BrdU-labelled tracks in cells treated with siMus81 in Fig. 2C). This could be explained by the fact that, although MUS81 depletion significantly reduced DNA damage produced by Chk1 inhibition or depletion, it did not reverse the stabilisation of Cdc25A caused by Chk1 inactivation (Fig. S3). Thus, MUS81 depletion does not appear to affect the increased CDK activity that is the main cause of reduced replication-fork progression associated with Chk1 deficiency [6], [24].

MUS81-depleted cells complete replication in the absence of an active Chk1, arguing against a model where replication failure in Chk1-inhibited cells is due primarily to increased replication-fork stalling. Instead, it is tempting to speculate that MUS81 can process replication forks into DSBs when Chk1 is inactive because of the dramatic decrease in replication-fork progression observed upon Chk1 inhibition. While fully active and processive replication forks might not be effectively targeted by MUS81 because of their dynamicity [13], the “slowed down” replication forks observed upon Chk1 inhibition could represent more suitable MUS81 substrates. MUS81-dependent collapsed forks that cannot be re-started when Chk1 is inactive would then be the main cause of incomplete replication.

A corollary of the above conclusions is that Chk1 activity protects replication forks from MUS81, and this could help explain why replication forks stalled by HU or aphidicolin are processed into DSBs only after prolonged drug treatments [23]. Thus, in such situations, initial Chk1 activation would prevent MUS81 from processing the forks, probably to promote a DSB-independent, Rad51-dependent fork restart [25]. However, as cells undergoing persistent replicative stress progressively inactivate Chk1 by degradation [26], the ensuing reduction in Chk1 activity would then allow MUS81 to collapse forks to promote DSB-mediated fork repair [23], [25].

While one possibility is that Chk1 directly controls MUS81 by mechanisms similar to those reported in fission yeast [15], we have not observed changes in MUS81 chromatin association or sub-cellular localization upon HU or AZD7762 treatments (Fig. S4). Furthermore, although Chk1 can phosphorylate MUS81 *in vitro* (Fig. S5A), we have been unable to identify Chk1-dependent phosphorylations on Mus81 or Eme1 *in vivo* (unpublished data) or detect effects of Chk1 on MUS81 nuclease activity (Fig. S5B). In light of these findings, it may be that Chk1 controls protein-protein interactions needed for MUS81 to exert its functions, or prevents remodelling of replication forks to create structures suitable for MUS81 activity.

Our data have established that, while replication-fork progression is severely impaired in the absence of Chk1 activity, Chk1 inactivation only compromises S-phase progression in the short term if forks can be collapsed by MUS81. Consequently, the absence of MUS81 results in decreased DSB formation, increased replication-fork progression and increased cell survival in Chk1-deficient cells. These effects of MUS81 depletion in cells acutely inhibited for Chk1 probably cannot, however, be extrapolated to scenarios where Chk1 is chronically inhibited or absent, due to other essential roles for Chk1, such as during mitosis [3], [27]. Nevertheless, it is noteworthy that the only metazoan cells reported to survive *CHK1* gene deletion are chicken DT40 cells [28], which lack a MUS81 ortholog [14, and our unpublished data]. Finally, we note that it will be of interest to determine whether MUS81 function/dysfunction influences how normal and cancer cells respond to Chk1-targeting drugs that are being developed as anti-cancer agents.

## Materials and Methods

### Human cell lines, transfection and siRNAs

Cells were grown in DMEM supplemented with 10% foetal bovine serum (FBS), penicillin, streptomycin, and glutamine. Human U2OS osteosarcoma cells (ATCC number HTB-96) were used throughout. Transfections were with Lipofectamine RNAiMAX (Invitrogen), and unless otherwise stated, experiments were performed 48 h afterwards. Protein extracts were prepared by lysis of cells in 2× Lämmli buffer and analyzed by SDS-PAGE. siRNA sequences: siLuc 5′-cguacgcggaauacuucga-tt-3′, siMus81#1 5′-gggaaggaagcuaagauccu-tt-3′, siMus81#2 5′-caggagccaucaagaauaa-tt-3′, siEme1#1 5′-accuaccuuuggcauuuaa-tt-3′, siEme1#2 5′-ggaaacagggagcaaauaa-tt-3′, siExo1 (and GFP-HA-Exo1 construct; 18). siChk1, and siChk2 were with siGENOME SMARTpool siRNA (Dharmacon). Antibodies used for western blots: Chk1 (1∶100 mouse G4; Santa Cruz), Chk2 (1∶250 rabbit; Abcam), Eme1 (1∶500 mouse; Santa Cruz), GFP (1∶1000 mouse; Roche), H2AX (1∶10 000 rabbit; Abcam), γH2AX (1∶1000 mouse; Upstate), KAP1 (1∶500 rabbit; Santa Cruz), KAP1 phospho-Ser-824 (1∶1000 rabbit; Bethyl), Mus81 (1∶1000 mouse; Abcam).

### Immunofluorescence

Cells were grown on poly-L-lysine coated coverslips, fixed with 2% paraformaldehyde for 10 min and permeabilized with 1× phosphate buffered saline (PBS) containing 0.2% (v/v) Triton X-100 for 5 min. Primary antibody staining was performed for 1 h in 5% FBS in 1×PBS (BrdU (1∶1000 mouse; GE Healthcare), γH2AX (1∶250 rabbit; Cell Signalling)). Secondary antibody staining was done with goat anti-mouse Alexa Fluor 488 or goat anti-rabbit Alexa Fluor 594 (1∶1000; Molecular Probes) for 30 min. Coverslips were washed 3× with 1×PBS and mounted on slides with Vectashield solution (Vector Labs) containing 4′,6-diamidino-2-phenylindole (DAPI) to stain DNA. All incubations were done at room temperature.

### DNA-fibre spreads

Were performed as described in [29]. BrdU was visualized with a primary antibody from BD Biosciences (1∶100, mouse) and a goat anti-mouse Alexa Fluor 594 secondary (1∶200; Molecular Probes).

### Flow cytometry

BrdU incorporation was measured with APC BrdU Flow Kit (BD Pharmingen) following manufacturer's instructions. Cells were pulsed with 10 µM BrdU 15 min before harvesting. γH2AX was detected using a mouse primary antibody (1∶100; Upstate) and a goat anti-mouse Alexa Fluor 488 secondary (1∶500; Molecular Probes).

### Neutral comet assays

Comet assays were with the Single Cell Gel Electrophoresis Assay-kit (Trevigen). Briefly, cells were trypsinized, resuspended in Mg^2+^- and Ca^2+^-free PBS, and counted. Approximately 1×10^6^ cells were mixed with low-melting agarose in a 1∶10 ratio, of which 75 µl was transferred onto Gel Bond film and covered with a 22 mm coverslip. Samples were incubated at 4°C in the dark for 30 min to solidify. Coverslips were removed and cells were lysed by incubation with lysis solution for 60 min at 4°C. Film slides were subsequently washed in TBE and run for 7 min at 35 volts on a horizontal electrophoresis apparatus in TBE buffer. Afterwards, film slides were fixed in 70% (v/v) ethanol for 5 min and allowed to dry overnight. DNA was visualized with SYBR-green dye and pictures were taken with a standard Olympus epifluorescence microscope.

Supporting Materials and Methods can be found in File S1.

## Supporting Information

Figure S1 **MUS81 depletion alleviates the S-phase progression defects associated with Chk1 deficiency.** Flow cytometry of replicating cells as measured by EdU incorporation. The x-axes show DNA content by propidium iodide (PI) staining; the y-axes represent EdU incorporation as measured by the EdU detection method. Graphics show representative images for each experiment. Insets show histograms obtained from the same samples. Percentages were calculated from three independent experiments (± SEM). Plots and quantifications were with FlowJo 9.0.2 software (Tree Star). Cells were transfected with siLuc or siMus81 #2 and then transfected with siChk1 as in Fig. 1D (*A*) or treated with 2 µM CEP-3891 for 12 h (*B*).
(TIF)

Figure S2 **MUS81 depletion reduces DNA double-strand break formation caused by Chk1 inhibition.** Pulse-field gel electrophoresis shows that MUS81 depletion abrogates DNA breakage after Chk1 inhibition. Cells were transfected as in Fig. 2, and treated with 200 nM AZD7762 for the indicated times (h). Intact genomic DNA does not enter the gel, while broken DNA migrates into it. Cells were treated with 5 µM etoposide (ETP; a DNA topoisomerase II inhibitor) for 3 h as a positive control for DNA double-strand break formation. Lambda phage DNA (M1) and yeast chromosomes (M2) were used as DNA markers.
(TIF)

Figure S3 **MUS81 depletion does not affect Cdc25A stabilisation caused by Chk1 inactivation.** Western blot analysis of cells transfected and treated as in Fig. 2A (*A*) or transfected with siMus81 and siChk1 as in Fig. 3C (*B*).
(TIF)

Figure S4 **Mus81 localization does not change upon DNA damage caused by hydroxyurea (HU) or AZD7762 treatments.** *A.* Chromatin fractionation shows no changes in Mus81 localization upon treatment with HU. Tubulin, DNA topoisomerase II beta, and histone H2AX were used as markers for cytoplasmic (C), nuclear (N2), and chromatin (P) fractions, respectively. Cells were treated with 2 mM HU for the indicated times. Antibodies recognizing RPA32 phosphorylated on Ser-4/8 were used to assess DNA-damage after HU treatment. *B.* Mus81 sub-cellular localization does not change upon Chk1 inhibition. Cells were transfected with pcDNA3-3×HA-Mus81, and 48 h afterwards were left untreated or treated with 200 nM AZD7762 for 5 h. Soluble proteins were pre-extracted with 1× phosphate buffered saline containing 0.2% (v/v) Triton X-100 prior to fixation. γH2AX antibodies were used to localize DNA-damaged cells.
(TIF)

Figure S5 **Chk1 kinase activity does not affect Mus81/Eme1 nuclease activity.** *A.* Chk1 phosphorylates Mus81/Eme1 *in vitro*. Coomassie staining of the purified Mus81/Eme1 complex and autoradiography upon kinase assay with purified Chk1 and γ-^32^P-ATP are shown. *B.* Autoradiography of nuclease assays performed on 3′-flap substrates. The Mus81/Eme1 site of DNA cleavage is indicated by an arrow. The star indicates the position of the radioactive label. The processed product runs faster in the gel than the substrate. Prior to addition of the DNA substrate, Mus81/Eme1 was subjected to a kinase reaction as in A.
(TIF)

File S1 Supporting Materials and Methods and Supporting References.
(DOC)
