# Supplementary figures and images for: Structure-Specific DNA Endonuclease Mus81/Eme1 Generates DNA Damage Caused by Chk1 Inactivation
Source: PLoS One. 2011 Aug 17;6(8):e23517. doi: 10.1371/journal.pone.0023517 (PMC3157403; doi:10.1371/journal.pone.0023517)

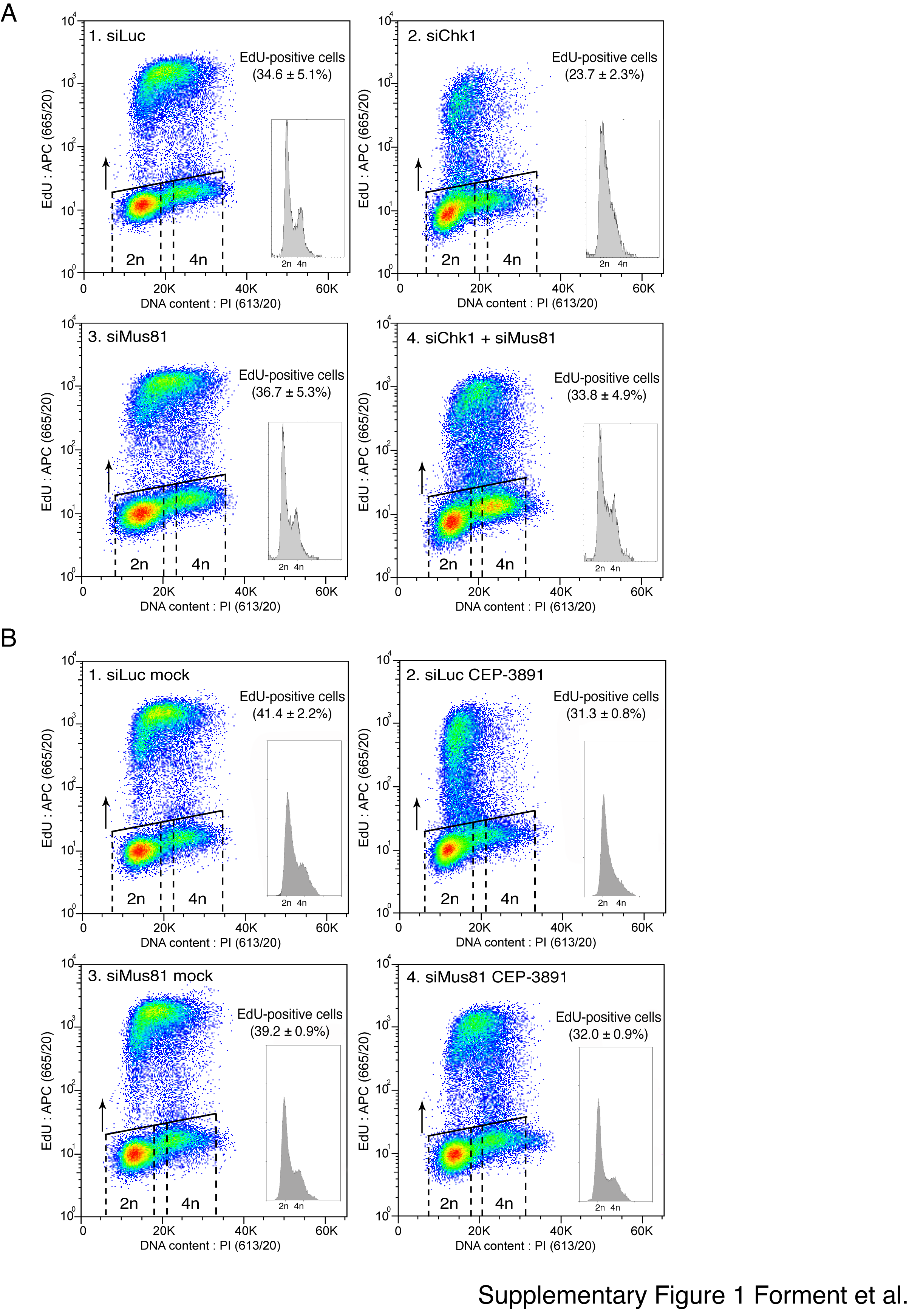

Supplement: Figure S1 — MUS81 depletion alleviates the S-phase progression defects associated with Chk1 deficiency. Flow cytometry of replicating cells as measured by EdU incorporation. The x-axes show DNA content by propidium iodide (PI) staining; the y-axes represent EdU incorporation as measured by the EdU detection method. Graphics show representative images for each experiment. Insets show histograms obtained from the same samples. Percentages were calculated from three independent experiments (± SEM). Plots and quantifications were with FlowJo 9.0.2 software (Tree Star). Cells were transfected with siLuc or siMus81 #2 and then transfected with siChk1 as in Fig. 1D (A) or treated with 2 µM CEP-3891 for 12 h (B). (TIF) [file pone.0023517.s001.tif]

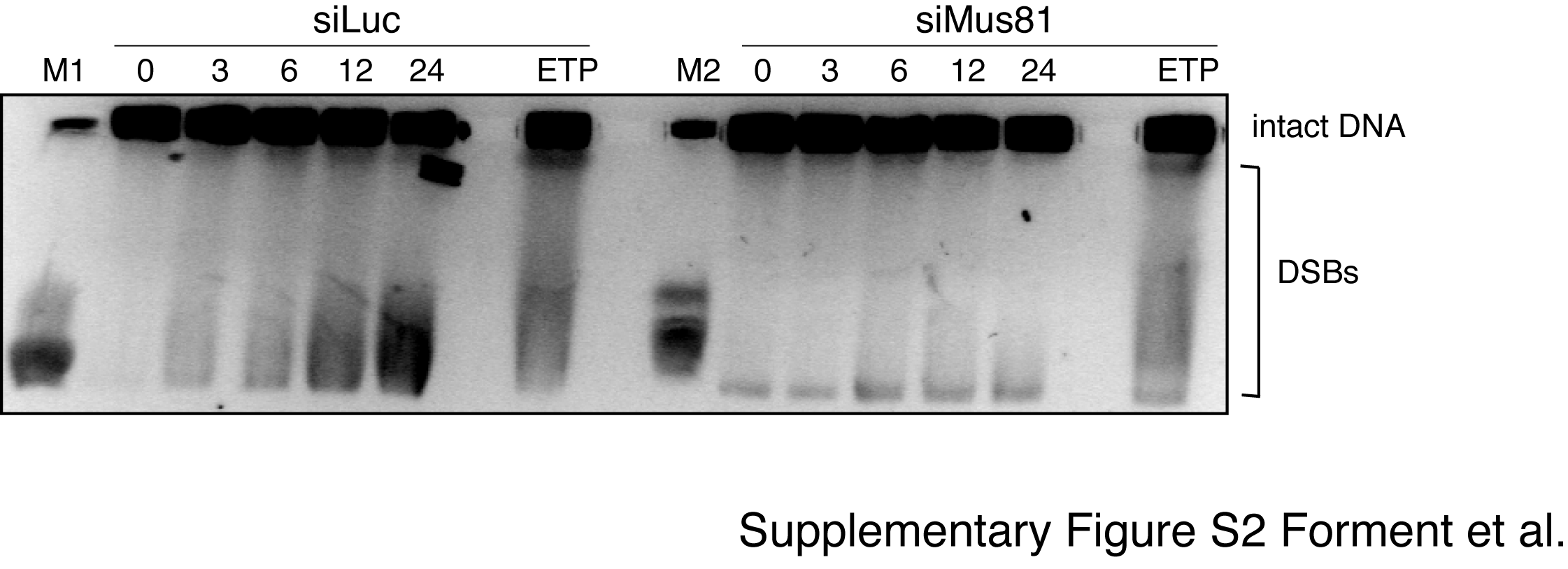

Supplement: Figure S2 — MUS81 depletion reduces DNA double-strand break formation caused by Chk1 inhibition. Pulse-field gel electrophoresis shows that MUS81 depletion abrogates DNA breakage after Chk1 inhibition. Cells were transfected as in Fig. 2, and treated with 200 nM AZD7762 for the indicated times (h). Intact genomic DNA does not enter the gel, while broken DNA migrates into it. Cells were treated with 5 µM etoposide (ETP; a DNA topoisomerase II inhibitor) for 3 h as a positive control for DNA double-strand break formation. Lambda phage DNA (M1) and yeast chromosomes (M2) were used as DNA markers. (TIF) [file pone.0023517.s002.tif]

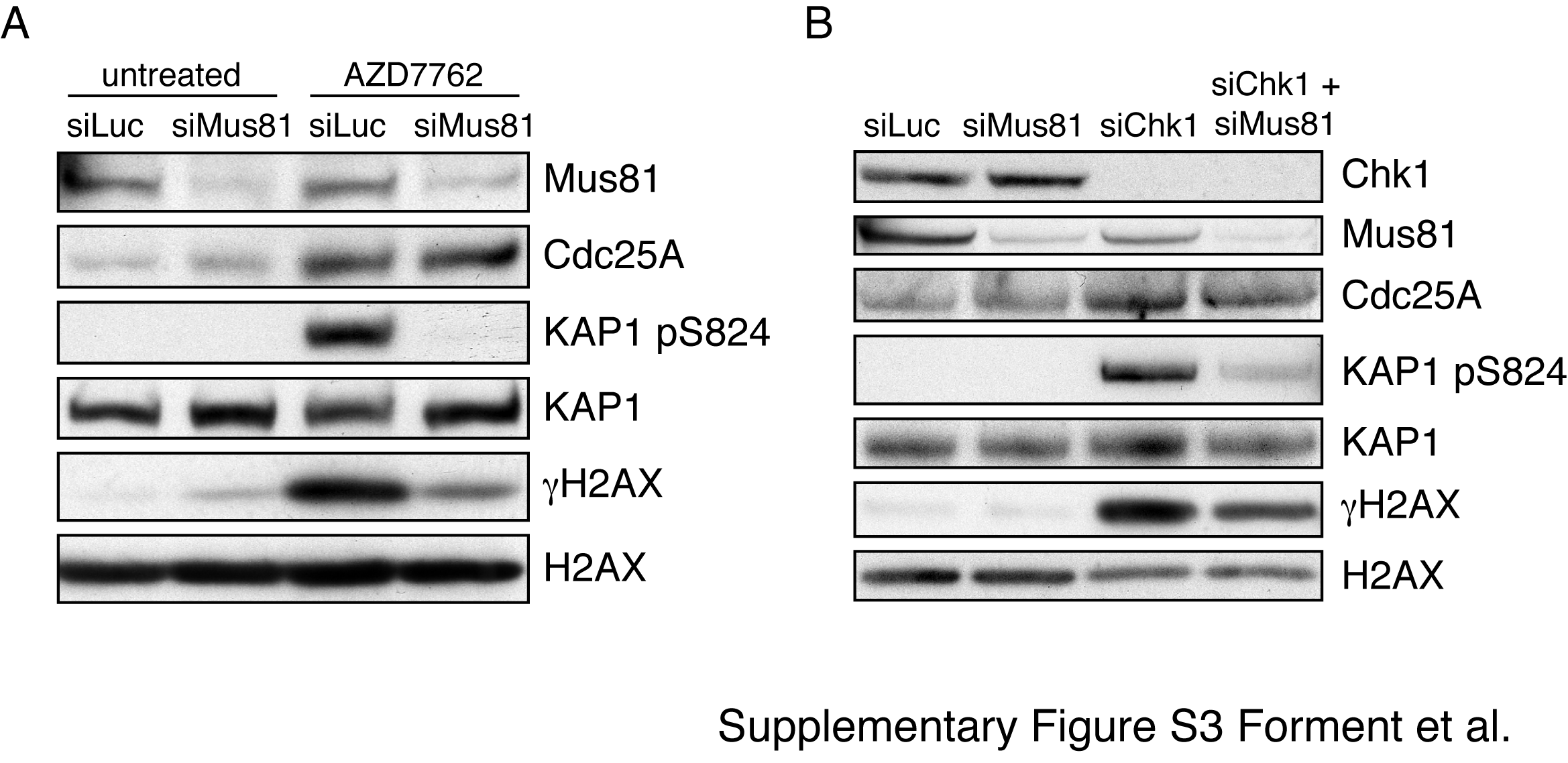

Supplement: Figure S3 — MUS81 depletion does not affect Cdc25A stabilisation caused by Chk1 inactivation. Western blot analysis of cells transfected and treated as in Fig. 2A (A) or transfected with siMus81 and siChk1 as in Fig. 3C (B). (TIF) [file pone.0023517.s003.tif]

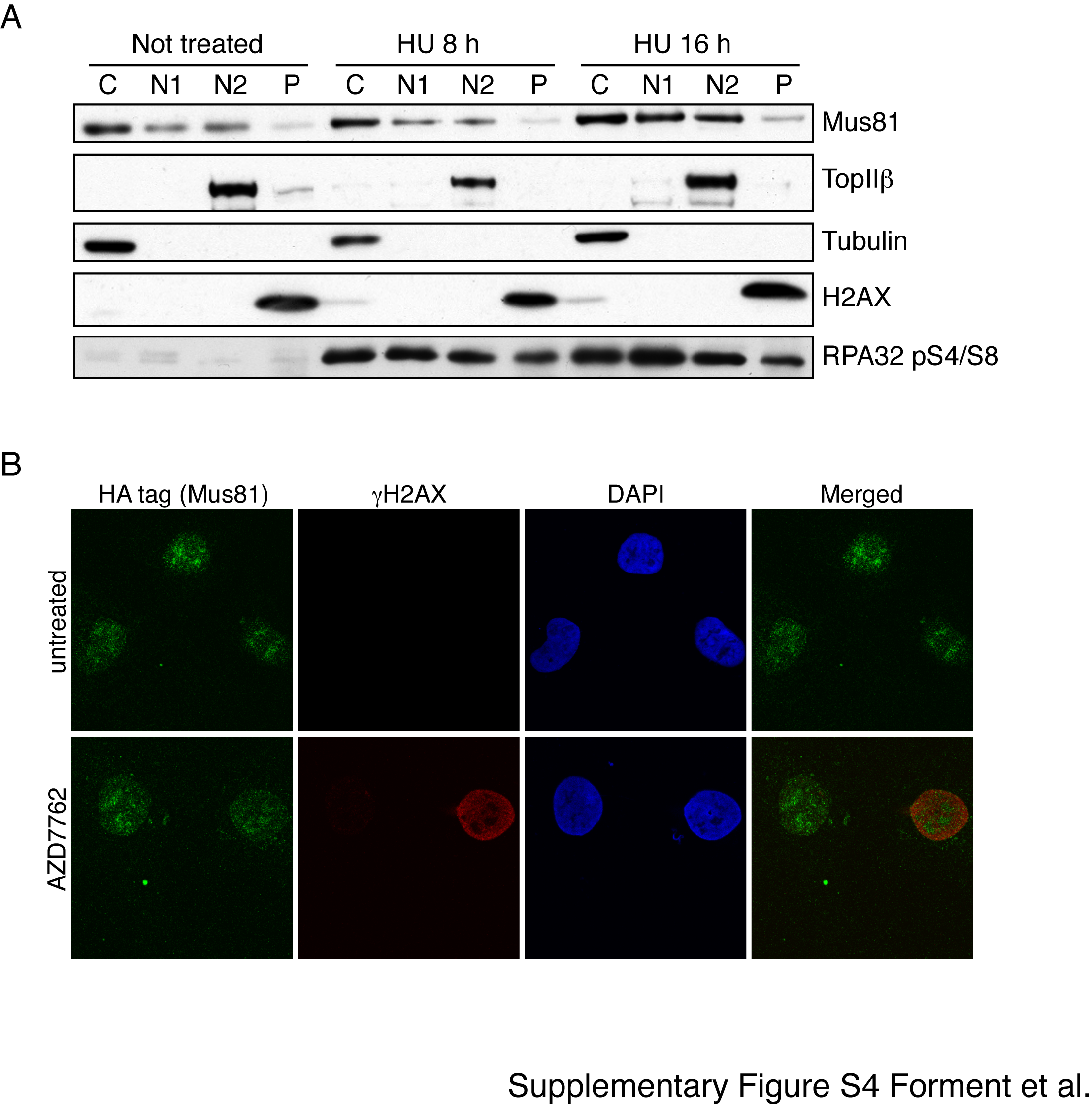

Supplement: Figure S4 — Mus81 localization does not change upon DNA damage caused by hydroxyurea (HU) or AZD7762 treatments. A. Chromatin fractionation shows no changes in Mus81 localization upon treatment with HU. Tubulin, DNA topoisomerase II beta, and histone H2AX were used as markers for cytoplasmic (C), nuclear (N2), and chromatin (P) fractions, respectively. Cells were treated with 2 mM HU for the indicated times. Antibodies recognizing RPA32 phosphorylated on Ser-4/8 were used to assess DNA-damage after HU treatment. B. Mus81 sub-cellular localization does not change upon Chk1 inhibition. Cells were transfected with pcDNA3-3×HA-Mus81, and 48 h afterwards were left untreated or treated with 200 nM AZD7762 for 5 h. Soluble proteins were pre-extracted with 1× phosphate buffered saline containing 0.2% (v/v) Triton X-100 prior to fixation. γH2AX antibodies were used to localize DNA-damaged cells. (TIF) [file pone.0023517.s004.tif]

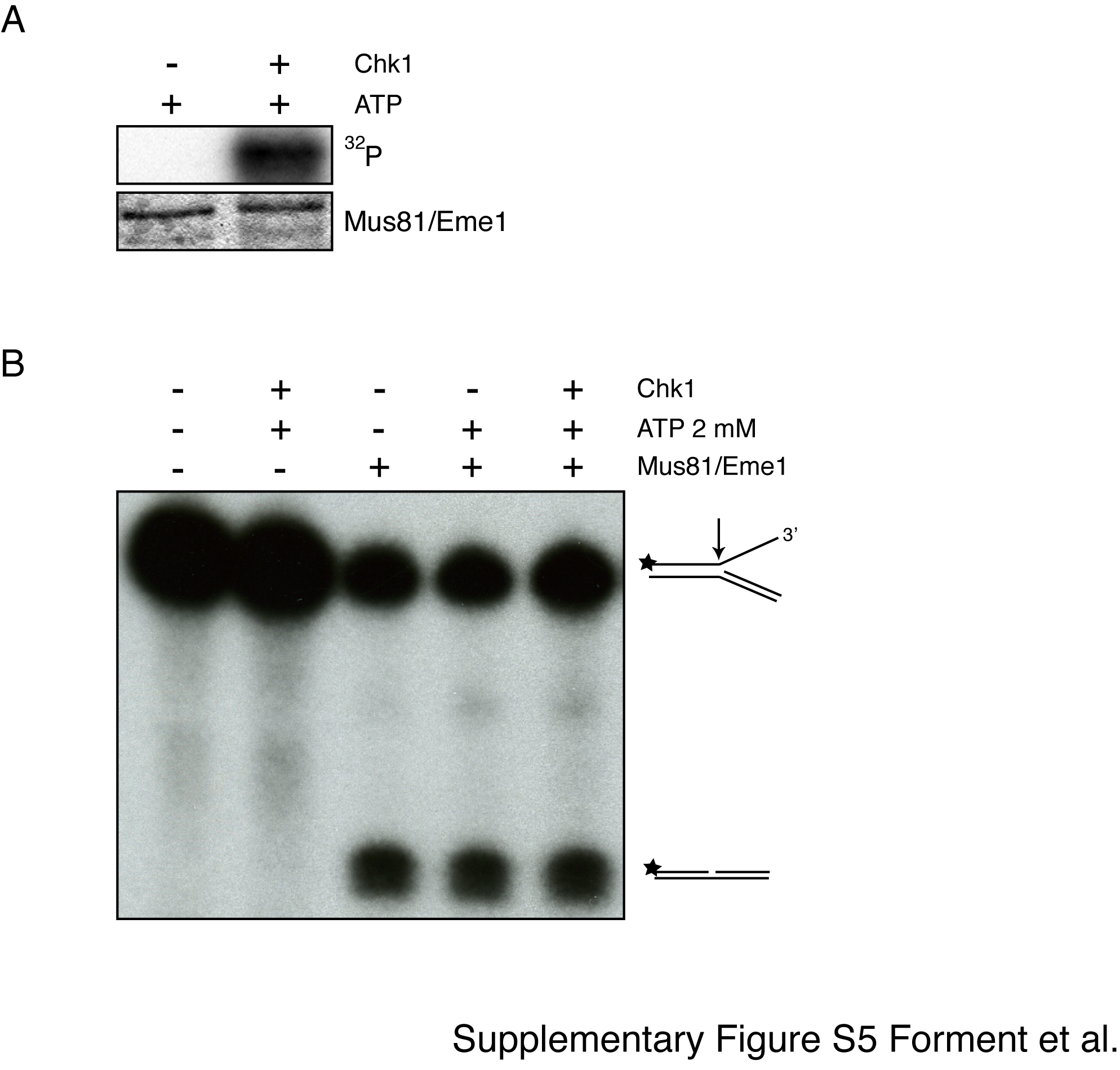

Supplement: Figure S5 — Chk1 kinase activity does not affect Mus81/Eme1 nuclease activity. A. Chk1 phosphorylates Mus81/Eme1 in vitro. Coomassie staining of the purified Mus81/Eme1 complex and autoradiography upon kinase assay with purified Chk1 and γ-32P-ATP are shown. B. Autoradiography of nuclease assays performed on 3′-flap substrates. The Mus81/Eme1 site of DNA cleavage is indicated by an arrow. The star indicates the position of the radioactive label. The processed product runs faster in the gel than the substrate. Prior to addition of the DNA substrate, Mus81/Eme1 was subjected to a kinase reaction as in A. (TIF) [file pone.0023517.s005.tif]
