# Supplementary material for: Structure-Specific DNA Endonuclease Mus81/Eme1 Generates DNA Damage Caused by Chk1 Inactivation
Source: PLoS One. 2011 Aug 17;6(8):e23517. doi: 10.1371/journal.pone.0023517 (PMC3157403; doi:10.1371/journal.pone.0023517)
Supplement: File S1 — Supporting Materials and Methods and Supporting References. (DOC) [file pone.0023517.s006.doc]

**Supporting Information**

**Structure-Specific DNA Endonuclease Mus81/Eme1 Generates DNA Damage Caused by Chk1 Inactivation**

Josep V. Forment, Melanie Blasius, Ilaria Guerini and Stephen P. Jackson

Contains Supporting Materials and Methods and Supporting References.

**Materials and Methods**

*Flow cytometry-* EdU incorporation was measured with Click-IT EdU Alexa Fluor 647 Flow Cytometry Kit (Invitrogen) following manufacturer’s instructions. Cells were pulsed with 10 μM EdU 30 min before collection.

*Pulse-field gel electrophoresis-* Cells were harvested by trypsinization and 10^7^ cells per sample were prepared in 1% (w/v) low melting-point agarose plugs. Solidified plugs were treated in a 0.5 M EDTA, 1% sarkosyl solution containing 0.4 mg/ml of proteinase K (Fluorochem) at 50 ºC overnight. Plugs were then left at 4 ºC for 1 h and washed three times with 1x Tris-EDTA pH 8.0. Plugs were loaded into a 1.5% agarose gel prepared in 0.5x Tris-borate EDTA, and sealed with 1% low melting-point agarose. Electrophoresis was on a Gene Navigator system (Amersham) at 50 V, 50-5000 s linear switch time for 66 h. DNA was stained with ethidium bromide.

*Chromatin fractionation-* Cells were scraped in 1xPBS and pellets were resuspended in Buffer A-10 (10 mM HEPES pH 7.4, 1.5 mM MgCl_2_, 10% glycerol, sucrose 0.5 mM, EDTA-free protease inhibitor cocktail (Roche), 0.1% Triton X-100, 10 mM KCl) and incubated 5 min on ice. Nuclei were harvested at 1,300 *g* 10 min 4 °C, and supernatant was collected as cytoplasmic fraction (C). Nuclei were resuspended in Buffer A-200 (Buffer A-10 with 200 mM KCl and without Triton X-100) and incubated 30 min 4 °C with constant shaking. Insoluble material was precipitated at 15,000 *g* 15 min 4 °C, and supernatants were collected as first nuclear fractions (N1). Insoluble material was resuspended in Buffer A-400 (Buffer A-200 with 400 mM KCl) and incubated 30 min 4 °C with constant shaking. Insoluble materials (chromatin pellets) were precipitated at 15,000 *g* 15 min 4 °C, and supernatants were collected as second nuclear fractions (N2). Chromatin pellets (P) were resuspended in 2x Lämmli buffer, boiled for 5 min, and syringed with a 25G needle. Equal amounts of each protein fraction were analyzed by SDS-PAGE.

*DNA transfection and antibodies-* Antibodies used for western blots: Cdc25A (1:100 mouse, Santa Cruz), DNA topoisomerase II beta (1:1000 rabbit, Santa Cruz), tubulin (1: 20 000 mouse, Sigma-Aldrich), H2AX (1:10 000 rabbit, Abcam), Mus81 (1:1000 mouse, Abcam), RPA32 phospho-Ser4/Ser8 (1: 10 000 rabbit, Bethyl). Antibodies used on immunofluorescence: HA tag (1:500 mouse, Covance), γH2AX (1:250 rabbit, Cell Signalling). Plasmid DNA was transfected using FuGENE 6 reagent (Roche) and following manufacturer’s instructions. pcDNA3-3xHA-Mus81 was a gift of Prof. C.H. McGowan (The Scripps Research Institute, La Jolla, CA).

*Protein purification-*Chk1 ORF was cloned into pFastBac1-TEV-SBP (generously provided by P. Marco-Casanova, Gurdon Institute) via EcoRI and XbaI restriction sites. Bacmids were prepared in DH10Bac™ *E.coli* cells (Invitrogen) following the manufacturer’s protocol. Chk1 was expressed and purified to homogeneity from Sf9 insect cells using the SBP tag (1). Mus81/Eme1 was expressed as a complex in bacterial cells using pET21d-Mus81/His:Eme1 (a gift of Dr. S. West, London Research Institute, Clare Hall), and purified as described previously (2).

*Kinase/nuclease assays-*The 3’-flap structure used as substrate for the nuclease assays was produced by annealing of oligos 1 (5'-GACGCTGCCGAATTCTACCAGTGCCTTGCTAGGACATCTTTGCCCACCTGCAGGTTCACCC-3'), 2 (5'-CATGGAGCTGTCTAGAGGATCCGACTATCGA-3'), and 3 (5'-ATCGATAGTCGGATCCTCTAGACAGCTCCATGTAGCAAGGCACTGGTAGAATTCGGCAGCGT-3') as previously described (3). Oligo 1 was 5'-^32^P-end-labelled. Prior to nuclease assays, preparations were subjected to a Chk1 kinase assay. Briefly, samples were prepared in kinase/nuclease buffer (50 mM Tris-HCl pH 7.5, 1 mM DTT, 7.5 mM MgCl_2_) and incubated in the presence/absence of 2 mM ATP and 1 µg of purified Chk1 for 10 min at 30 °C. Immediately afterwards the DNA substrate was added to the reaction and incubated for 30 min at 30 °C with constant agitation. Cleavage products were analyzed in a 12% denaturing PAGE.

**References**

1. Keefe AD, Wilson DS, Seelig S, Szostak JW (2001) One-step purification of recombinant proteins using a nanomolar-affinity streptavidin-binding peptide, the SBP-Tag. Protein Expr Purif 23: 440-446.
2. Ciccia A, Constantinou A, West SC (2003) Identification and characterization of the human mus81-eme1 endonuclease. J Biol Chem 278: 25172-25178.
3. Constantinou A, Chen XB, McGowan CH, West SC (2002) Holliday junction resolution in human cells: two junction endonucleases with distinct substrate specificities. Embo J 21: 5577-5585.
